# Reliability of psychiatric diagnoses in the 21^st^ century

**DOI:** 10.3389/fpsyt.2026.1891625

**Published:** 2026-07-29

**Authors:** Mateo Boberg, Mads Gram Henriksen, Jonas Berge, Nikolas Fascendini, Martin Jandl, Hanna Karakuła-Juchnowicz, Luís Madeira, Daniele Rossi Grauenfels, Hedda Soloey-Nilsen, Ana Giurgiuca, Igor Studart, Guilherme Messas, Tamara Ojeda Uribe, Luis Varela, Ricardo Corral, Michel Cermolacce, Tudi Gozé, Léon Franzen, Stefan Borgwardt, Jeff Huarcaya-Victoria, Konstantina Karkala, Ramune Mazaliauskiene, Jonas Eberhard, Troels Schmidt, Jean-Arthur Micoulaud-Franchi, Borut Skodlar, Julie Nordgaard

**Affiliations:** 1 Psychiatry East, Central and Western Zealand Hospital, University Hospital of Copenhagen, Roskilde, Denmark; 2 Department of Clinical Medicine, University of Copenhagen, København N, Denmark; 3 Center for Subjectivity Research, Department of Communication, University of Copenhagen, København S, Denmark; 4 Department of Psychiatry, Lund University, Lund, Sweden; 5 Department of Psychiatry, Clinica Santa Croce, Orselina, Switzerland; 6 Translational Research Center, University Hospital of Psychiatry and Psychotherapy, University Psychiatric Services (UPD) Bern, Bern, Switzerland; 7 Department of Psychiatry, Psychotherapy and Early Intervention, Medical University of Lublin, Lublin, Poland; 8 Lisbon School of Medicine, University of Lisbon, Lisbon, Portugal; 9 Dipartimento Salute Mentale – Dipendenze Patologiche, Azienda USL di Bologna, Bologna, Italy; 10 Department of Mental Health and Addiction Medicine, Nordland Hospital Trust, Bodø, Norway; 11 Department of Psychiatry, ‘Carol Davila’ University of Medicine and Pharmacy, Bucharest, Romania; 12 Department of Psychiatry, ‘Prof. Dr. Alexandru Obregia’ Clinical Psychiatric Hospital, Bucharest, Romania; 13 Department and Institute of Psychiatry, Universidade de São Paulo, São Paulo, Brazil; 14 Department of Psychiatry, University of Campinas, Campinas, Brazil; 15 Collaborating Centre for Values−Based Practice, St. Catherine’s College, University of Oxford, Oxford, United Kingdom; 16 Hospital Puerto Montt Dr. Eduardo Schútz Schroeder, Puerto Montt, Chile; 17 Facultad de Medicina, Universidad Andrés Bello, Santiago, Chile; 18 Servicio de Salud Mental y Psiquiatría, Hospital CRS El Pino, Servicio de Salud Metropolitano Sur, Santiago, Chile; 19 Teaching and Research Department, Hospital Borda, Universidad de Buenos Aires, Buenos Aires, Argentina; 20 Departamento de Docencia e Investigación, Fundación para el Estudio y Tratamiento de las Enfermedades Mentales (FETEM), Ciudad de Buenos Aires, Argentina; 21 Assistance Publique – Hopitaux de Marseille, University Department of Psychiatry, Marseilles, France; 22 Institut des Neurosciences de la Timone, CanoP Research Team, UMR 7289, Aix−Marseille Université, Marseille, France; 23 Pôle de Psychiatrie, Centre Hospitalier Universitaire de Toulouse – Université de Toulouse, Toulouse, France; 24 Équipe de Recherche sur les Rationalités Philosophiques et les Savoirs (EA 3051), Université de Toulouse, Toulouse, France; 25 Translational Psychiatry, Department of Psychiatry and Psychotherapy, University of Luebeck, Luebeck, Germany; 26 Escuela Profesional de Medicina Humana, Universidad Privada San Juan Bautista, Filial Ica, Peru; 27 Adult Psychiatric Department, General Panarkadian Hospital of Tripoli “Evangelistria”, Tripoli, Greece; 28 Department of Psychiatry, Lithuanian University of Health Sciences, Kaunas, Lithuania; 29 Department of Psychiatry, Skåne University Hospital, Helsingborg, Sweden; 30 Psykiatrisk vård Stockholm, Stockholm Health Care Services, Stockholm, Sweden; 31 Univ. Bordeaux, SANPSY, CNRS, UMR 6033, CHU de Bordeaux, Bordeaux, France; 32 Universitaire de Médecine du Sommeil, CHU de Bordeaux, Place Amélie Raba Léon, F-33000 Bordeaux, France; 33 University Psychiatric Clinic Ljubljana, Ljubljana, Slovenia; 34 Medical Faculty, University of Ljubljana, Ljubljana, Slovenia

**Keywords:** clinical vignettes, diagnostic accuracy, diagnostic reliability, ICD-10, inter-rater agreement, Krippendorff’s alpha, psychiatric diagnosis, psychopathology

## Abstract

**Background:**

Reliable diagnosis is a cornerstone of medical practice, yet concerns about diagnostic reliability have long haunted psychiatry. In the 1970s, the US–UK Diagnostic Project revealed striking international discrepancies in diagnostic practice. Although operationalized diagnostic criteria were introduced to improve reliability, contemporary evidence indicates that substantial problems persist. To investigate this, we conducted a large-scale international study of diagnostic reliability among medical doctors using standardized clinical case vignettes.

**Material and methods:**

In this cross−sectional study, medical doctors working in adult psychiatry across 19 countries from two continents assigned ICD−10 diagnoses to written cases. Each doctor was randomly assigned two of nine available cases. Inter−rater reliability among medical doctors was assessed using Krippendorff’s *α*. Diagnostic variability at case-level was quantified using Shannon entropy. We also calculated diagnostic accuracy as concordance with predefined best-estimate reference diagnoses.

**Results:**

A total of 1,038 medical doctors provided 1,902 diagnostic assessments across nine cases. Inter−rater reliability among medical doctors was modest (Krippendorff’s *α* = 0.48), indicating that diagnostic agreement between two doctors occurred in roughly 55% of cases. Overall diagnostic accuracy was 66%, with the lowest accuracy observed for cases depicting schizophrenia spectrum disorders. Diagnostic disagreements followed systematic patterns, with schizophrenia cases frequently misclassified as OCD, personality disorder, or bipolar disorder.

**Conclusions:**

Diagnostic inter−rater reliability among medical doctors remains limited, even under standardized conditions. The observed patterns of misclassification suggest that the variability in diagnostic assessments reflects systematic differences in clinical interpretation rather than random error, pointing to challenges in assessing psychopathology as well as ambiguities in psychiatric classification.

## Introduction

The success of modern medicine rests on the foundation of the medical paradigm, which relies on valid and reliable diagnoses. Procedures that ensure accurate diagnoses are essential for clinical decision-making and for research into treatment and prevention. Conversely, procedures that fail to ensure accurate diagnoses may hamper patient care and accumulation of scientific knowledge.

Fifty years ago, the US–UK diagnostic project shook the foundation of psychiatry. The study demonstrated an unacceptably low inter-rater reliability of psychiatric diagnoses—American psychiatrists diagnosed schizophrenia at far higher rates than their British colleagues. At the same time, the latter were more likely to diagnose affective disorders (1, 2). Striking as they were, these results were not unique but followed a pattern of studies documenting low diagnostic reliability dating back to the 1950s (3). To improve the inter-rater reliability of psychiatric diagnoses, i.e., the degree to which clinicians agree on patients’ diagnoses, the subsequent editions of the diagnostic manuals, i.e., the DSM-III (4) and ICD-10 (5) embraced the ‘operational revolution’ and introduced polythetic diagnostic criteria for mental disorders and rule-based classifications (6), thereby changing the diagnostic practice in psychiatry (7, 8).

Prior to DSM-III, studies using diagnostic criteria and structured interviews showed mixed effects on reliability, with improvements for some mental disorders but not for others (3, 9). The DSM-III field trials (4) subsequently reported much higher levels of diagnostic reliability, particularly when diagnoses were evaluated at the level of diagnostic groups rather than specific subtypes. Possibly due to the success of DSM-III, only few studies have since evaluated diagnostic reliability in clinical practice, and it has generally been assumed, rather than systematically demonstrated, that earlier times problems with diagnostic reliability had been largely fixed (6, 10). This assumption seems to rest more on semantics than on facts, i.e., there has been a notable shift in the threshold for what counts as “acceptable” inter-rater reliability (11). Whereas Spitzer and Fleiss (3) in the 1970s interpreted only kappa values above 0.7 as “satisfactory”, a standard echoed in the DSM-III field trials (4), the DSM-5 field trials interpreted kappa values in the range 0.40–0.59 as “good” and in the range 0.60–0.79 as “very good” (12). Looking at facts, however, a growing body of evidence challenges the assumption that diagnostic reliability has improved (11–13). Most notably, the DSM-5 field trials reported low diagnostic reliability for many mental disorders—with scores of inter-rater reliability (Cohen’s kappa values) comparable to those reported *prior* to the DSM-III field trials (12). See **Table 1** for the exact kappa-values from selected key studies.

**Table 1:** Inter-rater reliability (Cohen’s Kappa) from previous studies.

Diagnosis	Spitzer & Fleiss (3)	Helzer et al. (9)	DSM-III field trials (4)	DSM-5 field trials (12)
Overall	—	—	0.72	—
Schizophrenia	0.57	0.58	0.81	0.46
Mania/Bipolar I	0.41*	0.82	0.83	0.56
Depression		0.55		0.28
Anxiety/GAD	0.45	0.76	0.72	0.20

* Disorders of mania and depression were grouped together with other affective disorders under a broader *“affective disorders”* category in the DSM-III field trials and in the Spitzer & Fleiss study.

Taken together, these observations suggest that the question of diagnostic reliability in psychiatry remains unresolved. What, then, is the inter-rater reliability of psychiatric diagnoses in the 21^st^ century? To address this question, we conducted the first large-scale, cross-continental study of inter-rater reliability of ICD-10 psychiatric diagnoses using clinical case vignettes.

Prior studies of diagnostic inter-rater reliability have used direct patient interviews (1, 4, 12), videotaped interviews (2), and written clinical cases (14, 15). Direct interviews most closely resemble clinical practice but are difficult to standardize across large international studies, whereas video-based assessments preclude interaction and clarification. Written clinical cases allow identical clinical material to be assessed by large multilingual groups of clinicians under standardized conditions. Although written case-based assessments are more removed from routine clinical encounters, studies suggest that clinicians respond to well-constructed written cases in ways that resemble real-life clinical decision-making, making findings from written case-based studies comparable to those using patient interviews (16). For these methodological reasons, we applied written clinical cases in our study. Psychiatrists, psychiatric residents, and other medical doctors working in adult psychiatry across Europe and South America were asked to assign ICD-10 diagnoses to written clinical cases. This study design enabled a primary evaluation of inter−rater reliability and a secondary assessment of diagnostic accuracy measured as concordance with best-estimate reference diagnoses.

## Materials and methods

### Study design and participants

This cross-sectional study employed an online survey distributed across 19 countries officially using the ICD-10 (15 in Europe: Denmark, France, Germany, Greece, Italy, Lithuania, Norway, Poland, Portugal, Romania, Slovenia, Spain, Sweden, Switzerland, and United Kingdom; and 4 in South America: Argentina, Brazil, Chile and Peru) to examine inter-rater reliability of ICD-10 psychiatric diagnoses. Eligible participants were medical doctors working in adult psychiatry, including psychiatrists, residents, and other medical doctors. Recruitment was coordinated by local collaborators, who distributed the survey through their professional networks. Because of the wide distribution strategy, it was not possible to determine the ratio of invited doctors taking part in the survey.

### Survey design

Participants provided demographic and professional information before being randomly assigned two of nine written clinical cases. Participation was anonymous. The survey was conducted using the platform “SurveyMonkey” (17). For each case, the participant was asked to select an ICD-10 diagnosis from a predefined list of mental disorders. We restricted the diagnostic options from the more than 200 distinct mental disorders and subcategories available in the ICD-10 to a list of 30 mental disorders, which are commonly used in clinical practice and largely cover the spectrum of mental disorders (see **Supplementary Table 1S** in the **Supplementary Material**). This restriction served two purposes. First, it made the task more manageable and realistic for a survey format. Second, it increased the feasibility of analysis by avoiding an unworkable degree of fragmentation in the allocated diagnoses. We did not aim to capture the full granularity of ICD-10, but to assess the reliability of the main diagnostic categories represented in the cases. The survey and cases were translated by local psychiatrists. Minor contextual modifications were made, such as adapting case elements related to the local education and healthcare systems to fit the country in question. The survey was open for three months in 2025.

### Case vignettes

We developed seven written cases to resemble the kinds of clinical presentations that psychiatrists encounter in everyday practice. Each case was designed to meet the ICD-10 diagnostic criteria for a mental disorder. Two additional cases (Case 3 and 6) were adapted from the adult cases in the ICD-10 Casebook (18). Including these two cases allowed comparison between participants’ assessments of standardized ICD-10 cases and the newly developed cases. If diagnostic agreement differed markedly between the two types of cases, this could indicate problems related to the construction of the new cases in this study. It was not feasible to rely primarily on the ICD-10 Casebook cases as these had been publicly available for many years, and some participants could be familiar with them, potentially biasing diagnostic assessments. The seven cases were developed by three authors (MB, MGH, and JN) in collaboration with senior colleagues with expertise in the relevant diagnostic areas. For these cases, the best-estimate reference diagnoses were established through consensus and confirmed through independent review by senior psychiatrists with expertise in psychopathology. The best-estimate reference diagnoses (**Table 2**) are based on ICD−10 criteria, and the rationale for their selection is described in detail in the **Supplementary Material**. They are intended as clinically informed points of comparison rather than a definitive ground truth. Importantly, the evaluation of inter-rater reliability was conducted independently of the best-estimate reference diagnoses.

**Table 2:** Diagnostic accuracy: concordance with best-estimate reference diagnosis^*^.

Case	Best-estimate reference diagnosis	Concordance with best-estimate reference diagnosis
1	Schizophrenia	0.37 [0.31–0.44]
2	Schizophrenia	0.43 [0.36–0.50]
3	Schizophrenia	0.54 [0.47–0.60]
4	Schizotypal disorder	0.38 [0.32–0.45]
5	Bipolar disorder	0.94 [0.90–0.97]
6	Depressive disorders	0.88 [0.83–0.92]
7	Anxiety disorders	0.73 [0.66–0.79]
8	OCD	0.92 [0.88–0.96]
9	Personality disorders	0.72 [0.65–0.78]
All (weighted mean)		0.66 [0.64–0.68]

Values in brackets represent 95% confidence intervals calculated using Wilson score intervals.

The nine cases included three cases of schizophrenia (two of paranoid subtype, one of hebephrenic subtype), and single cases of schizotypal disorder, bipolar disorder, depressive episode, generalized anxiety disorder, obsessive–compulsive disorder (OCD), and emotionally unstable personality disorder. The number and composition of cases were determined pragmatically. We aimed to include a sufficiently broad range of common psychiatric disorders while keeping the survey manageable for participants and ensuring an adequate number of ratings per case. The selected diagnoses represent major diagnostic categories frequently encountered in adult psychiatric practice. We included three schizophrenia cases because research suggests that schizophrenia pose significant diagnostic challenges (12, 15). In addition, using multiple cases reduced the likelihood that participants would infer the intended diagnoses from discussions with colleagues.

As described above, written cases do not fully capture the complexity of real-life diagnostic encounters, but they provide a standardized assessment context in which all participants receive identical information, and they can be translated without loss of information. This reduces variability related to interview style, patient disclosure, and contextual factors, allowing a focused assessment of inter-rater reliability and an exploration of diagnostic accuracy.

### Analysis

Diagnoses were defined at the level of diagnostic groups for some disorders (**Table 3**) and at the level of specific diagnoses for others. Using these broader diagnostic categories reflects a clinically meaningful approach, and this approach was used in all statistical analyses.

**Table 3:** Diagnostic groups for some of the included diagnoses.

Diagnostic group
• Organic disorders (F00 Alzheimer’s disease, F05 delirium and F06.2 organic delusional disorder)
• Schizophrenia (F20.0 paranoid schizophrenia and F20.1 hebephrenic schizophrenia)
• Bipolar disorder (F30 manic episode and F31 bipolar affective disorder)
• Depressive disorders (F32 depressive episode and F33 recurrent depressive disorder)
• Anxiety disorders (F40.0 agoraphobia, F41.0 panic disorder, and F41.1 generalized anxiety disorder)
• Somatoform disorders (F45.0 somatization disorder and F45.2 hypochondriacal disorder)
• Personality disorders (F60.0 paranoid personality disorder, F60.2 dissocial personality disorder, and F60.3 emotionally unstable personality disorder)
• Autism spectrum disorders (F84.1 atypical autism and F84.5 Asperger’s syndrome)

Analyses examined diagnostic reliability among medical doctors, case−level variability in diagnostic assessments, and diagnostic accuracy measured as concordance with best−estimate reference diagnoses. Inter-rater agreement was assessed using Krippendorff’s *α*, which accommodates multiple raters who contribute varying numbers of ratings per case and handles missing data appropriately (19). As raters only assessed two out of nine possible cases each, the data structure was necessarily incomplete. Cohen’s kappa, which often is used to assess inter-rater reliability, is not applicable in our study, since Cohen’s kappa is restricted to exactly two raters and complete data. Although Cohen’s kappa and Krippendorff’s *α* differ in computation, both are interpreted on the same conceptual scale, i.e., a value of 0 reflects no agreement beyond chance and a value of 1 reflects perfect agreement.

Chance agreement (P_e_) was calculated from the empirical distribution of diagnostic categories by summing the squared category proportions. Observed agreement (P_o_) was then obtained using Krippendorff’s formulation, which relates *α*, P_e_, and P_o_. As an independent verification, we also computed P_o_ empirically by comparing all available rater pairs across cases and calculating the proportion of exact diagnostic matches. Because Krippendorff’s *α* excludes certain incomplete rater–case combinations, small numerical differences between the analytic and empirical estimates of P_o_ are expected.

Following Krippendorff’s conventions, *α* values were interpreted as follows: ≥ 0.80 as acceptable agreement, 0.67–0.80 as tentative agreement, and < 0.67 as poor agreement (19). Differences in inter-rater reliability between psychiatrists, residents, and other medical doctors, as well as between rater groups with varying levels of clinical experience (0–4, 5–9, 10–14, and 15+ years), were evaluated using permutation tests with 10,000 random label permutations.

Diagnostic variability was quantified using Shannon entropy (in bits) within each case. Entropy reflects the degree of dispersion across diagnostic categories, with higher values indicating greater diagnostic disagreement. The weighted mean entropy was calculated by weighting case−specific entropies by their number of ratings, and its 95% confidence interval was obtained using a bootstrap procedure (10,000 resamples). To evaluate whether cases differed in diagnostic variability, we conducted pairwise comparisons of entropy. For each pair of cases, the difference in entropy (ΔH) was estimated using non-parametric bootstrap resampling (10,000 iterations). Percentile-based 95% confidence intervals were computed for each ΔH. A difference was considered statistically significant when the confidence interval did not include zero.

The secondary outcome, i.e., diagnostic accuracy, was defined as the proportion of concordant assessments relative to the predefined best-estimate reference diagnosis. For example, when a case’s best-estimate reference diagnosis was a schizophrenia subtype, any schizophrenia subtype selected by the participant was coded as concordant. These diagnostic groupings were also applied in the reliability analyses (see **Table 3**).

### Ethics

The seven original cases are fictitious, and any resemblance to actual persons is coincidental. The survey involved only anonymized responses from medical doctors and did not include any patient data. According to ethical guidelines, such studies are exempt from ethics committee approval.

## Results

Demographic information about the participants is presented in **Table 4**. A total of 1,038 medical doctors contributed to the survey: 639 were psychiatrists, 282 were psychiatric residents, and 117 were other medical doctors working in adult psychiatry. A total of 1,902 diagnoses were allocated to the nine cases. Countries varied substantially in the number of diagnostic assessments they were able to obtain, with some collecting hundreds and others far less.

**Table 4:** Participant demographics.

Category	Number (%)
Sex	
Female	576 (55.5%)
Male	455 (43.8%)
Not disclosed	7 (0.7%)
Position	
Psychiatrist	639 (61.6%)
Resident in psychiatry	282 (27.2%)
Other medical doctor	117 (11.3%)
Experience (years)	
0-4	341 (32%)
5-9	244 (23.5%)
10-14	137 (13.2%)
15+	316 (30.4%)
Continent	
Europe	822 (79.2%)
South America	216 (20.8%)
Completion	
1 case vignette	174 (16.8%)
2 case vignettes	864 (83.2%)
Total	1038 (100%)

### Inter-rater reliability

The overall diagnostic reliability for all medical doctors was Krippendorff’s *α* = 0.48 (CI: 0.46-0.51). Stratifying reliability into the three different groups of doctors, Krippendorff’s *α* was 0.50 (CI 0.47-0.53) for psychiatrists; 0.45 (CI 0.41-0.51) for residents; and 0.45 (CI 0.40-0.56) for other medical doctors. Differences in Krippendorff’s *α* between the three groups were small (all *Δα* ≤ 0.05) and not statistically significant in permutation tests (*p* = 0.10–0.95). Consistently, diagnostic reliability did also not differ significantly, when doctors were stratified into years of clinical experience (0–4, 5–9, 10–14, and 15+ years). Therefore, we pooled data from all medical doctors in the analyses.

To make the level of diagnostic agreement more interpretable, we examined how much agreement was due to chance and how much reflected true concordance: Chance agreement was 13.1%, indicating that two raters would assign the same diagnosis in roughly one out of eight cases purely by chance. Krippendorff’s *α* was 0.48, meaning that raters resolved 48.0% of the disagreement expected by chance. The corresponding observed agreement was 54.9%. The empirical pairwise comparison method yielded a similar observed agreement of 55.3%. Overall, both estimates converge and indicate that two medical doctors assigning diagnoses to the same written clinical case agree approximately half of the time.

**Figure 1:**
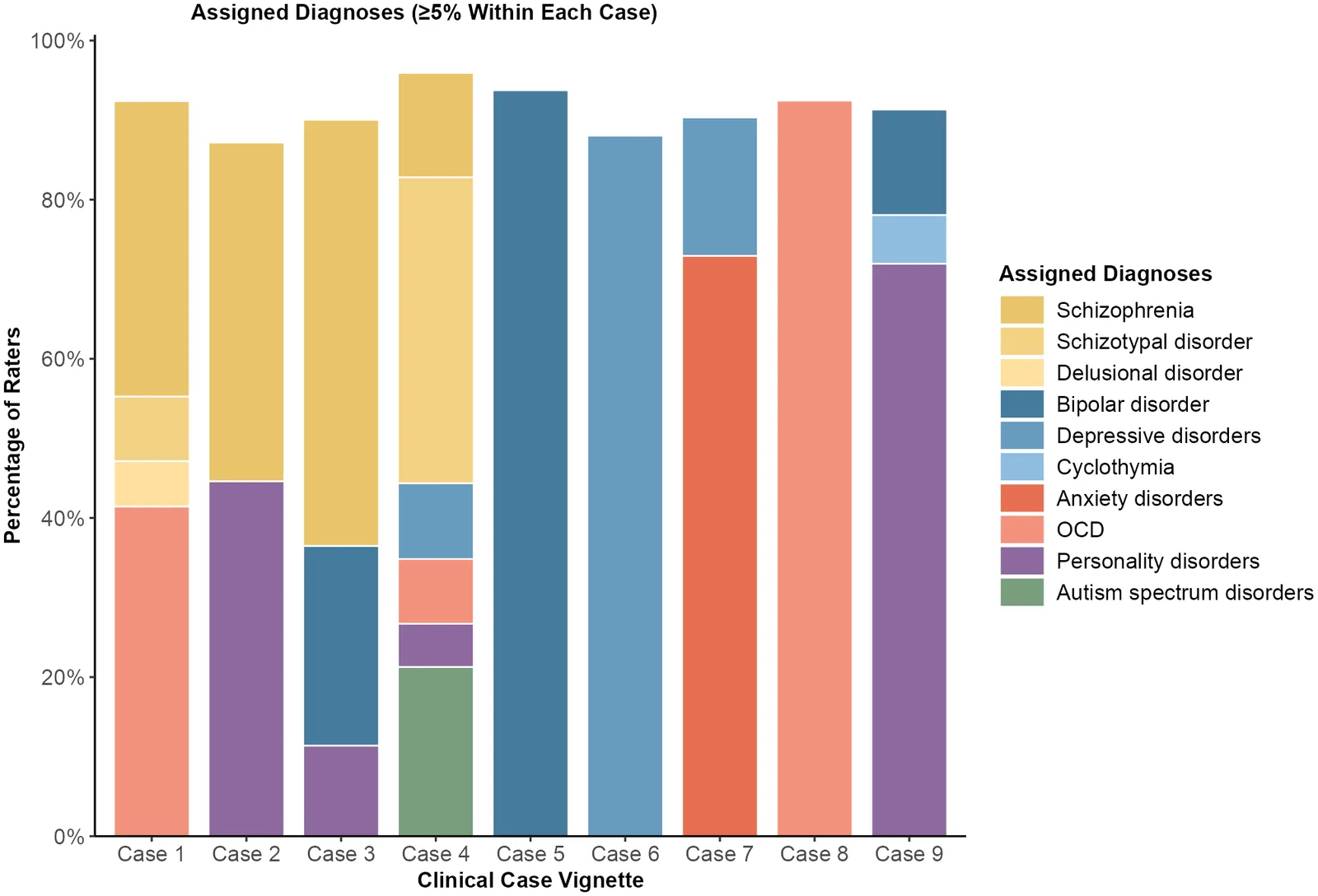
Assigned diagnoses (≥5% within each case).

**Figure 1** displays the most frequently assigned diagnoses (>5%) for each of the nine cases. Medical doctors showed marked variability across cases, with most variability observed for the cases reflecting schizophrenia spectrum disorders (Cases 1–4). Case 1 was mainly diagnosed as OCD (41%) or schizophrenia (37%). Case 2 was split between a personality disorder (45%) and schizophrenia (43%). Case 3 was most often diagnosed as schizophrenia (54%), followed by bipolar disorder (25%). Case 4 showed a wider spread, with schizotypal disorder (38%), autism spectrum disorders (21%), schizophrenia (13%), depressive disorders (10%), and OCD (8%). Cases 5 and 6 showed strong consensus: 94% diagnosed bipolar disorder for Case 5, and 88% diagnosed depressive disorders for Case 6. Case 7 was chiefly diagnosed as an anxiety disorder (73%), with 17% assigning a depressive disorder. Case 8 also showed strong agreement, with 92% diagnosing OCD. Case 9 was primarily diagnosed as a personality disorder (72%), with smaller proportions assigning bipolar disorder (13%) or cyclothymia (6%). Diagnostic allocations showed systematic patterns rather than random dispersion across categories. We did not observe any systematic difference in the assessment of the seven cases, which were developed for the study, compared to the assessment of the two cases adapted from the ICD-10 Casebook. All assigned diagnoses to the nine cases can be found in **Supplementary Table 1S** in the **Supplementary Material**.

**Figure 2:**
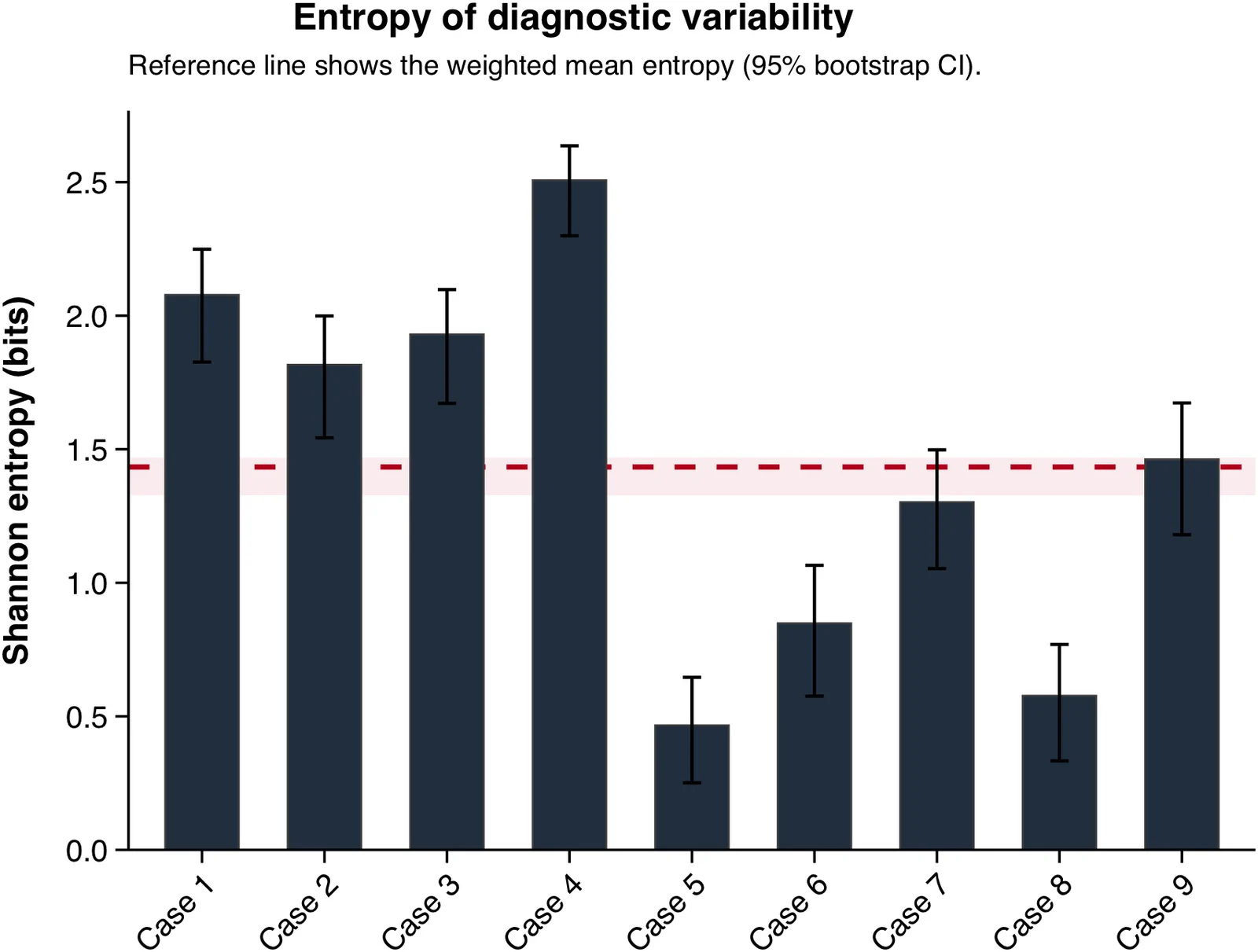
Entropy of diagnostic variability.

Entropy varied substantially across the nine cases (**Figure 2**). Case 4 had the highest entropy and differed significantly from all other cases. Cases 1–3 had the next highest entropy; they did not differ significantly from one another, but each differed significantly from Cases 5–9. Case 7 and 9 had significantly higher entropy than Case 5, 6, and 8. Case 7 and 9 did not differ significantly from each other. Case 5, 6, and 8 had the lowest entropy. Case 6 had significantly higher entropy than Case 5, and there was no significant difference between Case 5 and 8, and between Case 6 and 8, respectively. Overall, the cases with best−estimate reference diagnoses of schizophrenia−spectrum disorders (Cases 1–4) demonstrated markedly higher entropy than the remaining cases.

### Diagnostic accuracy: concordance with best-estimate reference diagnoses

Overall weighted diagnostic accuracy among medical doctors was 66% (see **Table 2**). Accuracy was lowest for Cases 1–4. The most frequent inaccurate diagnoses for Cases 1–3 (best-estimate reference diagnosis: schizophrenia) were OCD, personality disorder, and bipolar disorder, respectively. For Case 4 (best-estimate reference diagnosis: schizotypal disorder), the most frequent inaccurate diagnoses were autism spectrum disorders and schizophrenia. For Case 7 (best-estimate reference diagnosis: anxiety disorder), the most frequent inaccurate diagnosis was a depressive disorder. Concordance with the best-estimate reference diagnoses was highest for Cases 5, 6 and 8 (best-estimate reference diagnoses: bipolar disorder, depressive disorders, and OCD, respectively). However, these same diagnoses were also among the most common inaccurate diagnoses assigned to Cases 1–4 and 7, indicating problems in correctly allocating diagnoses of bipolar disorder, depressive disorders, and OCD to written cases despite the high diagnostic accuracy on cases with these disorders.

## Discussion

This international survey study provides the first large-scale, cross-continental assessment of the inter-rater reliability of psychiatric diagnoses using written cases. We found a poor overall reliability of psychiatric diagnoses (Krippendorff’s *α* = 0.48), corresponding to a situation in which two clinicians, assessing the same written clinical case, agree on the diagnosis approximately half of the time.

Comparison of this result with the DSM-III and DSM-5 field trials is limited by substantial methodological differences, including the use of live interviews rather than written cases, differences in study design, differences between DSM and ICD diagnostic systems, differences in the range of included mental disorders, and the application of different statistical measures of reliability. Still, the overall inter-rater reliability in our study on written clinical cases (Krippendorff’s *α* = 0.48) is notably below the Cohen’s kappa values reported for Axis I (*κ* = 0.72) and Axis 2 disorders (*κ* = 0.64) in the DSM-III field trials (4). The DSM-5 field trials include no overall inter-rater reliability scores, making a comparison with our result impossible. Still, the kappa values for several mental disorders in the DSM-5 field trials were much lower than those reported in the DSM-III field trials, indicating that our results may be more aligned with those of the DSM-5 field trials and the reliability studies preceding the DSM-III field trials (3, 9). Thus, our conclusion echoes that of Spitzer and Fleiss (3), who stated that the reliability of psychiatric diagnosis “is not good”. Despite the introduction of operational diagnostic criteria and rule-based classification, the overall reliability of psychiatric diagnosis, assessed on written cases, is modest.

It should be noted that although the cases were designed to contain sufficient information to assign ICD-10 diagnoses, participants were not explicitly instructed to systematically apply operational criteria when making their diagnostic judgments. Consequently, our findings may reflect the reliability of clinicians’ diagnostic judgments as they are typically made in routine clinical practice rather than the reliability of a strictly criterion-based diagnostic procedure.

While diagnostic accuracy, measured as concordance with best-estimate reference diagnoses, was high for some cases (i.e., OCD and affective disorders), it was substantially lower for schizophrenia spectrum disorders. A crucial observation is that the diagnostic disagreements observed in our study were not random. Rather, they followed systematic and clinically meaningful patterns. Cases 1–4, with best-estimate reference diagnoses of schizophrenia spectrum disorders, showed the highest entropy. These cases were frequently assigned other diagnoses, such as OCD, personality disorder, or bipolar disorder, while these same diagnoses showed high concordance with best-estimate reference diagnoses when presented in a more prototypical form (Cases 5, 8 and 9). This pattern suggests that clinicians are not simply guessing or confused but are applying different interpretive frameworks when evaluating complex clinical presentations. In other words, the observed diagnostic variability reflects structured diagnostic divergence rather than random error.

The observed diagnostic patterns point toward specific mechanisms underlying this variability. One contributing factor appears to be a selective weighting of symptom clusters. Clinicians often seemed to anchor on salient features, such as obsessive symptoms, while underweighting diagnostically decisive features, such as psychotic symptoms that would shift the diagnosis within the ICD-10 hierarchy. This is particularly evident in cases, where multiple symptom domains co-occur as, e.g., in schizophrenia spectrum disorders. In such cases, the hierarchical structure of ICD-10 requires that higher-ranked mental disorders take precedence (5), but this principle was not consistently applied. These findings suggest that the application of diagnostic criteria depends not only on their formal definition, but also on clinical judgment regarding symptom salience and relevance.

From a clinical perspective, the level of diagnostic agreement is not merely a statistical concern but has direct implications for patient care. If two clinicians are likely to assign different diagnoses to the same patient, this raises questions about the stability of treatment decisions, prognostic expectations, and communication across clinical settings. Diagnostic categories are often treated as if they reflect stable, clearly bounded entities, but the present findings suggest that their application in clinical practice may be more variable. Notably, diagnostic agreement did not differ significantly among the doctors, neither when stratified into position (psychiatrist, residents, and other medical doctor) nor when stratified into years of clinical experience. Thus, the observed diagnostic variability cannot be explained simply by varying levels of clinical experience.

Our written case survey differs from real-world clinical encounters in several important respects. On the one hand, written cases represent a relatively favorable condition for assessing diagnostic agreement, as all doctors received identical, preselected information deemed sufficient to assign an ICD-10 diagnosis. This removes several sources of variability inherent to clinical practice, including differences in interview technique, clinician focus, and patient disclosure. On the other hand, written cases omit important aspects of real clinical encounters, such as dynamic interaction, language, affect, and the possibility of clarifying symptoms and their temporal and contextual development. While these factors may, in some cases, improve diagnostic accuracy, the standardized nature of the present design suggests that the observed level of agreement may reasonably be considered an upper bound. This suggests that diagnostic reliability in routine clinical settings is likely to be lower than what we have reported here. Findings from previous studies, using either interviews with patients or videotaped interviews, do not demonstrate better levels of diagnostic inter-rater reliability, except for the DSM-III field trials more than forty years ago (**Table 1**).

These implications extend to psychiatric research. If diagnostic categories are applied inconsistently, research samples may be less homogeneous than assumed. This may obscure identification of disorder-specific mechanisms, hinder replication of research findings, and impede translation of research into effective treatments. The present findings may also help explain why symptoms frequently appear across diagnostic categories, and why transdiagnostic approaches have gained increasing attention.

The results of this study should be interpreted considering several limitations. First, although written case-based designs allow for standardized assessment of diagnostic agreement, they cannot capture the complexities of real clinical encounters. Second, the sample represents a voluntary and unevenly distributed group of clinicians across countries. Participation was based on professional networks and voluntary response, and response rates could therefore not be calculated. It is possible that participants had a greater interest in psychopathology, diagnosis, or academic psychiatry than clinicians, who chose not to participate. If so, the observed level of diagnostic agreement may represent a relatively favorable estimate of diagnostic reliability. Nonetheless, the international scope and diversity of the medical doctors strengthen the external validity of the findings, but the unequal distribution of participants across countries may have influenced the reported estimates. Third, grouping related ICD-10 diagnoses into broader diagnostic categories yielded higher diagnostic agreement estimates than would have been obtained with fully granular ICD-10 diagnoses (cf. **Supplementary Table 1S** in the **Supplementary Material**). Fourth, we also restricted the diagnostic options to 30 commonly used ICD-10 disorders. While this was necessary for feasibility and analysis, it may have increased agreement by reducing the range of available diagnostic alternatives. In addition, providing respondents with a predefined list of diagnoses may have influenced their diagnostic decision-making by prompting consideration of diagnoses that might not otherwise have been actively entertained. However, because the list consisted of commonly used psychiatric diagnoses routinely considered in clinical practice, the magnitude of such a checklist effect is likely to have been limited. Taken together, the observed inter-rater reliability may represent a more favorable estimate of diagnostic agreement compared to a situation where the full range of ICD-10 diagnoses is available.

Clinicians’ diagnostic judgments may have been influenced by cognitive biases such as confirmation bias, whereby an initial diagnostic impression affects the interpretation and weighting of subsequently presented information. Although all participants received identical information, differences in diagnostic reasoning may also reflect the cognitive processes involved in interpreting the information (20). Importantly, such cognitive biases are also likely to influence diagnostic decision-making in routine clinical practice.

In conclusion, despite decades of methodological refinement and the introduction of diagnostic criteria and rule-based classification, the inter-rater reliability of psychiatric diagnoses remains modest, even under the standardized conditions of written clinical cases. The present findings could suggest that refinement of diagnostic criteria alone may be insufficient for achieving high levels of diagnostic agreement. Further empirical research is needed to better understand the sources of diagnostic variability and how they may be addressed to improve clinical practice and psychiatric research.

## Data Availability

Datasets presented in this article are not readily available, as we are Currently preparing additional articles based on the dataset. Requests to access the datasets should be directed to matbo@regsj.dk.
