# Supplementary material for: Reliability of psychiatric diagnoses in the 21st century
Source: Front Psychiatry. 2026 Jul 29;17:1891625. doi: 10.3389/fpsyt.2026.1891625 (PMC13462468; doi:10.3389/fpsyt.2026.1891625)
Supplement: Supplementary file 1 [file SupplementaryFile1.pdf]

Supplementary Material

Table of Contents

Table 1S. The 30 diagnoses available for rating in the survey, and the diagnoses assigned to the nine cases *2*

Comment to Table 1S *3*

Cases and diagnostic reasoning *4*

Case 1. Paranoid Schizophrenia **5**

Case 2. Paranoid Schizophrenia **7**

Case 3. Hebephrenic Schizophrenia **10**

Case 4. Schizotypal Disorder **12**

Case 5. Bipolar Disorder **15**

Case 6. Depressive Episode **18**

Case 7. Generalized Anxiety Disorder **21**

Case 8. Obsessive–Compulsive Disorder **24**

Case 9. Emotionally Unstable Personality Disorder **26**

Table 1S. The 30 diagnoses available for rating in the survey, and the diagnoses assigned to the nine cases

	Case 1	Case 2	Case 3	Case 4	Case 5	Case 6	Case 7	Case 8	Case 9
**Diagnosis**	**N (%)**	**N (%)**	**N (%)**	**N (%)**	**N (%)**	**N (%)**	**N (%)**	**N (%)**	**N (%)**
F00 Dementia in Alzheimer's disease	--	--	--	--	1 (<1)	1 (<1)	1 (<1)	--	1 (<1)
F05 Delirium	--	--	--	1 (<1)	--	1 (<1)	--	--	--
F06.2 Organic delusional disorder	3 (1)	4 (2)	6 (3)	--	--	2 (1)	1 (<1)	--	--
F10 Substance use disorders	--	1 (<1)	1 (<1)	--	--	1 (<1)	--	--	1 (<1)
F20.0 Paranoid schizophrenia	**74 (35)**	**78 (40)**	69 (33)	18 (8)	1 (<1)	9 (4)	--	--	--
F20.1 Hebephrenic schizophrenia	4 (2)	5 (3)	**44 (21)**	11 (5)	--	1 (<1)	--	--	1 (<1)
F21 Schizotypal disorder	17 (8)	2 (1)	5 (2)	**85 (38)**	--	2 (1)	--	1 (<1)	2 (1)
F22.0 Delusional disorder	12 (6)	--	2 (1)	--	--	3 (1)	--	--	--
F30 Manic episode	--	--	18 (9)	--	25 (11)	--	--	--	--
F31 Bipolar affective disorder	1 (<1)	2 (1)	35 (17)	1 (<1)	**185 (83)**	4 (2)	2 (1)	1 (<1)	26 (13)
F32 Depressive episode	--	--	--	4 (2)	1 (<1)	**198 (88)**	33 (16)	--	--
F33 Recurrent depressive disorder	1 (<1)	1 (<1)	--	17 (8)	--	1 (<1)	3 (1)	4 (2)	--
F34.0 Cyclothymia	--	--	--	--	3 (1)	--	--	1 (<1)	12 (6)
F40.0 Agoraphobia	--	--	--	1 (<1)	--	--	13 (6)	--	--
F41.0 Panic disorder	--	--	--	--	--	--	79 (38)	1 (<1)	--
F41.1 Generalized anxiety disorder	5 (2)	--	--	3 (1)	--	1 (<1)	**59 (29)**	5 (2)	--
F42 Obsessive-compulsive disorder	87 (41)	--	1 (<1)	18 (8)	1 (<1)	1 (<1)	3 (1)	**196 (92)**	--
F43.1 Post-traumatic stress disorder	--	5 (3)	1 (<1)	--	--	--	2 (1)	1 (<1)	--
F43.2 Adjustment disorder	--	--	--	--	--	--	--	--	--
F44 Dissociative disorders	1 (<1)	4 (2)	1 (<1)	1 (<1)	--	--	--	--	--
F45.0 Somatization disorder	--	--	--	--	--	--	1 (<1)	--	--
F45.2 Hypochondriacal disorder	--	1 (<1)	--	--	--	--	9 (4)	--	--
F50.0 Anorexia Nervosa	--	--	--	--	--	--	1 (<1)	--	--
F60.0 Paranoid PD	1 (<1)	--	--	7 (3)	--	--	--	--	--
F60.2 Dissocial PD	--	3 (2)	--	--	--	--	--	--	1 (<1)
F60.3 Emotionally unstable PD	1 (<1)	84 (43)	24 (11)	5 (2)	7 (3)	--	--	1 (<1)	**140 (72)**
F60.31 Impulsive type	1 (<1)	12 (6)	10 (5)	--	1 (<1)	--	--	--	34 (17)
F60.32 Borderline type	--	72 (37)	14 (7)	5 (2)	6 (3)	--	--	1 (<1)	106 (54)
F84.1 Atypical autism	1 (<1)	4 (2)	1 (<1)	18 (8)	--	--	--	--	2 (1)
F84.5 Asperger's syndrome	1 (<1)	--	--	29 (13)	--	--	--	1 (<1)	2 (1)
F90.0 Hyperkinetic disorder	1 (<1)	1 (<1)	1 (<1)	2 (1)	--	1 (<1)	--	--	8 (4)
**Number of ratings for the case**	210	195	211	221	224	226	207	212	196

Numbers in bold indicate the *exact* best-estimate reference diagnosis. PD=Personality Disorder.

Comment to Table 1S

Table 1S shows all diagnoses assigned in the survey.

In Table 1S, the exact best-estimate reference diagnoses are highlighted in bold. These exact best-estimate reference diagnoses are also those suggested for the nine cases in the differential diagnostic considerations in the Supplementary Material.

While we believe that our use of broader diagnostic categories in the study (Table 3) reflects a clinically meaningful approach, it does increase the inter-rater reliability estimate compared with an analytic approach in which agreement is assessed at the level of the exact diagnoses - in the latter case, Krippendorff’s α is 0.40 (CI: 0.38–0.43).

Cases and diagnostic reasoning

The nine case vignettes included in the survey are presented below. Our arguments for assigning the study's best-estimate reference diagnoses are made with reference to the ICD-10 "Blue Book"[1]. For each case vignette, we have outlined the relevant diagnostic criteria to support our reasoning. Where appropriate, we have quoted short excerpts directly from the ICD-10 in italics.

We also offer considerations on the alternative diagnoses suggested by many respondents in the survey, and we describe why we don't find these to be the most appropriate diagnoses for the case vignettes.

[1] World Health Organization. 1992. The ICD-10 Classification of Mental and Behavioural Disorders. Clinical descriptions and diagnostic guidelines. World Health Organization: Geneva.

Case 1. Paranoid Schizophrenia

A 19-year-old female, Maria, was referred to psychiatric assessment by her general practitioner, who suspected OCD. Maria is an only child raised in a caring family. Her parents are both ambitious academics. Her father's brother is drinking too much alcohol. She lives with her parents in a nice house in a quiet and safe neighbourhood. She does well academically. Socially, she mostly keeps to herself and only has a few friends. She is currently in secondary school, but it has become increasingly difficult for her to concentrate on schoolwork and to show up for class. She has not attended classes for the last 6 months. Maria reports that she does not use substances.

Maria describes feeling anxious much of the time. It is difficult for her to be around many people or in crowds. In such situations, she becomes very aware of how she behaves and what others might think of her, and she feels unsafe. She also reports feeling anxious at school.

In the last couple of years, she has become increasingly occupied with taking a range of precautions to keep her safe. She is very afraid of what might happen to her during the night. She is afraid that snakes will enter the house and come to her room on the second floor. She is worried that the snakes might find a sharp object, such as a pencil, and poke it into her eyes. To make sure this does not happen, she must sweep the whole second floor for any sharp object every night before going to bed. This sweeping must be done exactly the same way every night. She is also afraid that she might hurt herself during the night by going down to the basement and drinking soap for the washing machine. She denies suicidal ideation, but she is afraid that she might unintentionally hurt herself in her sleep. To avoid this from happening, she must arrange the clothes she wore during the day in a specific way on the floor in her bedroom. If this is not done accurately, she must rearrange the clothes all over again. She spends a significant amount of time every night performing these activities. Due to these activities, she often ends up sleeping only a few hours and is too tired to go to classes.

The patient and her parents are very worried about her situation. The patient breaks into tears easily when talking about her situation, which she believes is dangerous for her. It is difficult for the psychiatrist to maintain eye contact with Maria during the interview. She explains that she avoids eye contact, because too much of her personal information is revealed through her eyes. The patient and her family report that Maria has exhibited this behaviour for several months.

ICD-10 Criteria

(a) *thought broadcasting*: Evidenced by the patient's avoidance of eye contact. Her explicit statement that personal information is revealed through her eyes indicates a belief in involuntary transmission of thoughts rather than a mere concern about vulnerability or social discomfort. The fact that she avoids eye contact demonstrates that the belief that personal information is being

revealed through her eyes has reality status for her. The patient and her family report that this behaviour has lasted for months.

(d) *persistent delusion* that is *culturally inappropriate and completely impossible*: Her belief that snakes wield sharp objects and may poke such objects into her eyes qualifies as such a “bizarre” delusion. Here, too, we notice that she acts upon this belief by sweeping the whole second floor for sharp objects before going to bed, demonstrating again the belief’s reality status for her. We are told that she, in the last couple of years, has been occupied with taking precautions to keep herself safe at night (sweeping the floor and arranging the clothes), and this takes a substantial amount of time every night. The “bizarre” delusion has thus been present for an extended period.

Maria meets the criteria for the paranoid subtype of schizophrenia. Her delusions are prominent, while disturbances in affect, volition, speech, and catatonic symptoms seem to be absent.

Diagnostic Considerations

If Maria’s ideas about the snakes and risk of involuntary suicide were to be classified as true obsessions, Maria would have to consider these ideas unreasonable and have inner resistance to them while experiencing them. This case vignette describes no such resistance nor that she finds the ideas unreasonable. By contrast, it is stated that Maria considers herself to be in a dangerous situation. Her sweeping the house for sharp objects is driven by the bizarre delusion.

It is important to note that patients with schizophrenia can present with both obsessive and compulsive symptoms. However, psychotic symptoms in general and bizarre delusions in particular are not part of the ICD-10 diagnosis of OCD. In the description of OCD in ICD-10, it is emphasised that patients often, unsuccessfully, try to resist the obsessive thoughts and compulsive acts, and this is reflected in criterion “*b*”, which states that *there must be at least one thought or act that is still resisted unsuccessfully, even though others may be present which the sufferer no longer resists.* Maria does not resist any thought or act in the vignette. Regarding the differential diagnosis to schizophrenia, ICD-10 states, *obsessional symptoms developing in the presence of schizophrenia, Tourette's syndrome, or organic mental disorder should be regarded as part of these conditions*.

Case 2. Paranoid Schizophrenia

Helena, a 21-year-old woman, was involuntarily admitted to the psychiatric hospital a month ago following a suicide attempt. She had written a farewell letter to her family, and she set the alarm clock with the intention of jumping out of the window upon awakening. However, the alarm did not wake her up, and the suicide was not carried out.

Developmentally, Helena progressed age-appropriately in motor and language skills, but she experienced stuttering as a child. She grew up with her parents and an older brother. The family home had a high level of conflict. Her mother works in homecare, and her father is a craftsman.

During childhood, Helena had a pronounced demand-avoidant behaviour and unstable affect. She also had difficulties maintaining friendships. She was intensively bullied, which led her to change schools several times. She graduated from school with below-average grades.

In tenth grade, Helena had several conflicts with her peers and teachers, and she was repeatedly caught lying. These incidents, along with other psychological difficulties, resulted in her leaving school before completing the grade. Since then, she has displayed a pattern of instability, and she has had frequent psychiatric hospitalisations for crises that have prevented her from participating in further educational or vocational programs.

From an early age, she had a reputation for fabricating stories, including allegations of violent assaults that could not be substantiated. At the age of 16, she deceived a young man into buying her expensive gifts.

Starting in her early teenage years, Helena had numerous contacts with the healthcare system due to physical complaints such as vomiting blood, fainting, and patellar luxations. None of which were observed by others, and no physical health problem was found despite numerous examinations.

Helena had two outpatient contacts in child and adolescent psychiatry, where it was noted that she copied others' behaviour, engaged in self-harm, and told untrustworthy stories. Intelligence testing revealed an IQ of 92, with an uneven profile and linguistic skills far below average.

She exhibits many different symptoms, including compulsions, anxiety, symptoms of an eating disorder, cutting, and suicidal thoughts. She also describes occasionally hearing voices, and she has been observed seemingly having visual hallucinations. She also reports feeling sometimes observed.

At the age of 20, Helena was seen a few times in the Psychiatric Emergency Unit, where she reported hearing a male voice speaking to her. She explained that this voice started talking to her after she was raped by a friend's older brother at the age of 11.

During a two-month admission, she elaborated on this experience. She described a male voice that spoke directly to her. It encourages her to self-harm and commit suicide. It also tells her that she is being watched and followed. She said that those following her always managed to hide. She constantly checks the room to find out where they are, looking in drawers, cupboards, behind furniture, doors, etc.

Helena added that her thoughts sometimes sound like a loud echo and are out of her control. In these situations, she is afraid others can hear her thoughts. She described herself as increasingly affected by lack of energy, difficulty getting out of bed, and a feeling of being overwhelmed.

During the psychiatric interview, Helena appeared friendly and accommodating, but other times evasive and irritable. She was described as having a neutral mood and normal psychomotor activity.

ICD-10 Criteria

(a) *thought echo* and *thought broadcasting*: Helena reports that her thoughts sometimes sound like a loud echo and are beyond her control, and in such situations she fears that others can hear her thoughts. However, it is described as occurring "sometimes," and it is unclear whether these symptoms meet the ICD-10 requirement of occurring most of the time over a period of 1 month or more.

(e) *persistent hallucinations in any modality, when accompanied either by fleeting or half-formed delusions without clear affective content, or by persistent over-valued ideas, or when occurring every day for weeks or months on end:* Helena has experienced regular auditory hallucinations for the past 10 years. Persecutory delusions often accompany the hallucinations.

(h) *"negative" symptoms*: Helena reports feeling "increasingly affected by lack of energy" and having "difficulty getting out of bed."

(i) *a significant and consistent change in the overall quality of some aspects of personal behaviour, manifest as loss of interest, aimlessness, idleness, a self-absorbed attitude, and social withdrawal*: Helena's condition is marked by aimlessness and withdrawal from education, work, and social life.

Helena's condition further qualifies for the paranoid subtype of schizophrenia, as the clinical picture is marked by auditory hallucinations, often accompanied by persistent delusions of being watched and followed.

Diagnostic Considerations

Helena exhibits long-lasting and pronounced psychotic symptoms. The only conceivable alternative to a diagnosis of a psychotic disorder would be the suspicion of symptom simulation.

Elements of her personal history, including a reputation for fabricating stories, unsubstantiated claims of assault, and unverifiable somatic complaints, could raise concerns about feigning. However, these features could just as well be understood as early manifestations of persecutory and somatic delusions rather than deliberate deception.

The ICD-10 outlines two forms of simulated psychiatric symptoms. The first is factitious disorder, which is categorised among adult personality disorders. In this condition, the simulation of symptoms is *best understood as a disturbance of illness behaviour and an adoption of the sick role.*

Although this possibility cannot be entirely excluded, as would be the case for all people, it appears unlikely in Helena's situation. Factitious disorder remains a rare disorder, whereas psychotic disorders are prevalent in psychiatric settings. Apart from a reputation for dishonesty, which itself may be subject to alternative interpretations, there is no evidence that Helena is engaging in factitious behaviour.

The second form of symptom simulation described in ICD-10 is malingering. This involves the intentional feigning of symptoms *motivated by external stresses or incentives*. According to ICD-10, malingering is considered *uncommon* outside legal contexts and requires a clearly identifiable external motive. The manual states that the motivation for malingering is *obvious and usually confined to situations where personal danger, criminal sentencing, or large sums of money are involved.* In Helena's case, there are no discernible external incentives that would support a sustained and purposeful fabrication of psychotic symptoms over several years. Importantly, neither factitious disorder nor malingering were among the possible options to rate in the survey.

Helena demonstrates interpersonal instability and has experienced significant conflict in relationships with teachers and peers. Although this could superficially resemble features of a personality disorder, the presence of persistent and clearly defined psychotic symptoms strongly supports a diagnosis of a primary psychotic disorder. These symptoms are not limited to moments of crisis but are consistently present over a decade and evidenced by her continual vigilance for perceived persecutors. According to ICD-10, personality changes are seen in severe mental disorders.

Case 3. Hebephrenic Schizophrenia

Silvia is a 23-year-old woman. She was compulsorily admitted to the psychiatric hospital after the neighbours had contacted the police as she had been screaming and throwing things out the window during a discussion with her parents.

Silvia's birth was a difficult forceps delivery. Childhood was also difficult as her father abused alcohol and could be violent when drinking. Her academic performance was good despite skipping school relatively often. Socially, she was well-liked, funny, and it was easy for her to make new friends. Silvia's parents described her as an outgoing person who could "talk to anyone". She has not been able to keep relationships for more than a couple of weeks. Silvia has had several sex partners, often with men much older than her. She lives with her parents, but she doesn't help with any house chores. Her mother cleans her room, prepares the food, etc. She has been unemployed most of the time since leaving school.

She has become increasingly anxious and agitated, and she often starts arguments with her parents. She occasionally sees shadows in the periphery of her vision and hears the doorbell ringing, but, upon checking, nobody is there. Recently, she told her parents that she was being persecuted, but a few days later this didn't seem to bother her, and she instead started talking about being "the chosen one". These and other peculiar ideas seem to come and go. She is talkative and her speech can be a bit difficult to follow.

When she was 21 years old, she was admitted to the psychiatric unit for the first time due to severe anxiety, sleeplessness, and aggressive outbursts toward others, primarily her mother. During the first hospitalisation she appeared agitated, had incoherent speech, and incongruous affect. She accidentally set herself on fire after pouring some paraffin over her bed and was burned over parts of her body. At the time of discharge, she had labile affect, and her behaviour seemed somewhat aimless and incoherent. She was referred to outpatient treatment, which she discontinued soon after.

At the time of the compulsory admission, Silvia appeared dishevelled. She was restless, and her behaviour was erratic. She shouted "abuse!" at the nurses and ran wildly around in the hospital corridors. On one occasion, she ate some flowers and drank the water from the vase they were in. She was observed to have rapidly shifting affect and agitation.

ICD-10 Criteria

(f) *breaks or interpolations in the train of thought, resulting in incoherence or irrelevant speech, or neologisms*: Silvia's speech is generally mildly disorganised. Yet, during her first hospitalisation, it was described as incoherent, illustrating a formal thought disorder. Although this criterion is most

likely fulfilled, it remains unclear whether this thought disorder has persisted at the required level of severity for the 1 month duration specified by ICD-10.

(h) "*negative symptoms such as marked apathy, paucity of speech, and blunting or incongruity of emotional responses, usually resulting in social withdrawal and lowering of social performance:* In Silvia's case, we see a notable lack of initiative in occupational and domestic activities as she has been unemployed since leaving school, does not contribute to household responsibilities, and relies entirely on her mother for basic tasks such as cleaning and food preparation.

(i) *a significant and consistent change in the overall quality of some aspects of personal behaviour, manifest as loss of interest, aimlessness, idleness, a self-absorbed attitude, and social withdrawal*: Despite early signs of sociability and academic ability, Silvia's current lifestyle is characterised by aimlessness, idleness, and erratic, disorganised behaviour. Though she may be emotionally volatile, her reactions lack meaningful direction or purpose and do not translate into sustained engagement with her environment. This is also evident through incongruous emotional responses, including labile and inappropriate affect.

Silvia also describes brief visual and auditory hallucinations and appears to experience fleeting delusions, though the ideas on "being the chosen one" and being persecuted are only very briefly mentioned in the case.

The clinical picture, characterised by disorganised behaviour including incoherent and disorganised speech, incongruous and labile affect, lack of goal-directed activity, and the absence of prominent catatonic symptoms or a sustained delusional system, supports the diagnosis of hebephrenic schizophrenia.

Diagnostic Considerations

The general criteria for schizophrenia (F20) are met by items (h) and (i), and most likely also by item (f). However, fulfillment of a third criterion is not formally required to establish the diagnosis. Additionally, several other features support this direction, including brief hallucinations and delusions, though these have been fleeting.

The patient does not meet the ICD-10 criteria for an affective disorder. Although she is described as exhibiting agitation and elevated psychomotor activity, there is no evidence of a clearly elevated mood or distinct, time-limited episodes with preserved functioning outside those periods. Silvia's unstable relationships, impulsivity, and emotional lability could superficially resemble a personality disorder. However, the presence of disorganised speech, bizarre behaviour (e.g., eating flowers and drinking the water from the vase they were in), and brief or fleeting hallucinations and delusions cannot be accommodated by a personality disorder, but point toward a primary psychotic disorder.

Case 4. Schizotypal Disorder

Darius is a 21-year-old man. He complains of generally feeling unwell during the day, with a slight improvement in mood in the late afternoon. He often feels tired, sleeps a lot, and tends to isolate. He does not find joy in anything but says that he doesn't feel sad either. He doesn't feel much at all. This is not new but has been so for several years.

At night, he often ponders various topics and replays daytime conversations in his mind, e.g., imagining scenarios where he is humiliated. He compares his thoughts to branches of a tree that keep branching out in ever new directions. His thoughts can be about a million different things, he says, and sometimes he simply cannot keep track of them. He also regularly ponders questions such as the meaning of life or words. He also entertains thought experiments about whether things which he cannot see actually are there (he knows that they are) and whether the colour red might appear as blue to someone else. He has been contemplating such questions since childhood. Sometimes, all this thinking makes him uneasy, and he tries to manage by playing computer games.

Darius feels that people look at him and talk about him, especially during stressful periods. He is highly vigilant about the actions of people around him, such as on a bus, and although he does not believe they want to harm him, he always remains watchful.

For a considerable time, Darius has been largely inactive, which he reports has reduced his level of stress. He lives in his father's basement. He has one friend, who accepts that Darius sometimes does not answer the phone for days, when he doesn't feel like talking.

He used to entertain the idea that he had to touch his eyebrows six times, whenever smoking a cigarette. He cannot explain why he did so but states he was a bit obsessed with his eyebrows, and at one point he had to touch his eyebrows every five minutes. He stopped doing this when others started commenting on it. Sometimes, he imagines himself hurting other people in a grotesque and very violent way. When these scenes pop up in his mind, he can't do anything but watch them until they pass. Even though he is repulsed by these thoughts, he is also a bit fascinated by them. He sometimes worries if he one day would carry out such violent acts, but he brushes this fear off as ridiculous.

In school, he did well academically but often failed to do his homework. Socially, he had difficulties, was bullied, and changed schools five times. Eventually, he became partially integrated into some social groups at school, but he seldom met up with friends outside of school. He reports feeling different from others and not quite knowing who he is. He dropped out of high school and worked for two years as a cleaner. He later worked at a kindergarten but left due to the overwhelming noise.

Darius first came into contact with psychiatric services at the age of 17, where he was suspected to suffer from depression. At age 19, he attempted suicide, and he has been on antidepressants since that time. He does not believe he suffers from depression and doesn't think the medication works.

During the interview at the outpatient clinic, he presented as friendly, well-groomed, and intelligent. His affect was somewhat flattened, and his mood was neutral. His answers to the questions were vague and sometimes tangential.

ICD-10 Criteria

(a) inappropriate or constricted affect: Darius presents with flattened affect and emotional detachment. He reports "not feeling much at all" and does not experience joy or sadness, suggesting emotional constriction.

(c) poor rapport with others and a tendency to social withdrawal: Darius lives in isolation in his father's basement, has only one friend, and often avoids contact for days. He reports longstanding difficulties with social integration.

(e) suspiciousness or paranoid ideas: Darius feels watched and talked about, especially during stressful periods. Though he does not believe others intend him harm, his hypervigilance and mistrust reflect paranoid ideation.

(f) obsessive ruminations without inner resistance, often with dysmorphophobic, sexual or aggressive contents: He experiences intrusive, violent thoughts that disturb him but also fascinate him. He cannot stop these scenes from unfolding in his mind, and while he finds them repulsive, he does not actively resist them.

(h) vague, circumstantial, metaphorical, over-elaborate, or stereotyped thinking, manifested by odd speech or in other ways, without gross incoherence: His speech is vague and tangential.

Diagnostic Considerations

Darius does not meet criteria for a psychotic disorder. His occasional feelings of being watched or talked about are brief, lack delusional conviction, and do not reflect a break from reality. He remains aware of their irrational nature. His vague and tangential speech is not severe enough to meet the criterion of *(f) breaks or interpolations in the train of thought, resulting in incoherence or irrelevant speech, or neologisms* (as defined in the criterion for schizophrenia). There are no hallucinations, no delusions, and the behavioural disorganisation is subtle.

Although the lack of joy and passivity could suggest a depressive disorder, the condition is not episodic. Still, it has been present over many years, with no identifiable periods of remission or return to baseline functioning. And it doesn't appear in relation to depressed mood. This persistent

course and lack of depressed mood, combined with constricted and limited affect, point away from a typical mood disorder and suggest a more pervasive disturbance.

While his social difficulties since childhood could align with autism spectrum disorder, there is no indication of the *restricted, repetitive, stereotyped patterns of behaviour, interests, and activities,* required for a diagnosis of Asperger's syndrome. Atypical autism is also not appropriate, as stated in ICD-10: *Atypical autism arises most often in profoundly retarded individuals whose very low level of functioning provides little scope for exhibition of the specific deviant behaviours required for the diagnosis of autism; it also occurs in individuals with a severe specific developmental disorder of receptive language.*

These clearly do not apply to Darius. He is neither profoundly intellectually disabled nor affected by a severe receptive language disorder. On the contrary, Darius was described as intelligent during the psychiatric interview, demonstrating intact language comprehension and abstract thinking, despite some vagueness and tangentiality in his speech. Moreover, according to ICD-10 Blue Book, *In most cases, development is abnormal from infancy and, with only a few exceptions, the conditions become manifest during the first 5 years of life* (p. 252). There is no description of Darius' social functioning at that young age. He did not encounter psychiatry before the age of 17, where there seems to have been a change in his condition.
The vivid inner pictures of hurting others are not true obsessions, since there is no inner resistance to these experiences as they appear. These experiences are thus rated as criterion (*f*) for schizotypal disorder as described above. Darius' feeling of needing to touch his eyebrow a certain number of times does not seem to be related to any specific thoughts about it, and he cannot describe a reason for doing it. Finally, he ceased the behaviour after others commented on it. Thus, this does not seem to be a compulsion and there is no obsessional thought connected to it.

Case 5. Bipolar Disorder

Elisa is a 37-year-old woman, who came to the psychiatric emergency unit with her spouse after self-harming by cutting her thigh. She reported feeling overwhelmed, and she described anxiety and low mood that had lasted weeks. Elisa has been on sick-leave from her job, where she was recently reassigned from a project manager role to an administrative position. She is married with two young children, aged 3 and 5, and the family has stable finances.

Elisa described feeling “emotionally unstable” for years, noting significant mood shifts that began in her mid-20s. She often feels either “up” or “down,” and she has difficulty recalling longer periods, where her mood was neutral. In her youth, she had frequent conflicts with teachers and authority figures, resulting in several school changes. Her spouse described her as “strong-willed” and noted she has, at times, acted impulsively and disregarded others’ needs.

In her young adult years, Elisa occasionally faced trouble with law enforcement for minor infractions such as shoplifting and driving without a license. She dismissed these incidents as “youthful mistakes” and downplayed their seriousness. She also mentioned a history of lying to get out of difficult situations, including fabricating excuses to avoid work or social commitments.

Over the past few months, Elisa reported increasing distress. Workplace changes left her feeling inadequate and unmotivated, and she often ended up merely staring at her computer. She struggled to fall asleep and frequently woke during the night. Her appetite declined, and she lost 6 kg. In desperation, she self-harmed by cutting her thigh but denied any intention to end her life, though she had thoughts about “wishing to escape everything.”

Elisa’s spouse described her as increasingly withdrawn, tearful, and unresponsive to attempts at reassurance. At the emergency room, Elisa avoided eye contact, responded slowly to questions, and appeared disorganized in her thoughts. She was admitted to the psychiatric unit but requested discharge within a week, stating that “people at the hospital were trying to control her.”

At her follow-up psychiatric consultation five weeks after discharge, Elisa’s spouse reported a dramatic change in her behaviour over the past weeks. Elisa described feeling energised and full of ideas. She had become highly active, engaging in multiple projects simultaneously, though some were abandoned before completion. She had also joined several online forums, reconnecting with old acquaintances and sharing personal stories, sometimes inappropriately.

Elisa also reported a newfound interest in physical activity, going on long runs late at night. Her spouse noted she was only sleeping 3-4 hours per night but seemed unaffected by the lack of rest. Elisa acknowledged feeling confident and creative, but she also had moments of irritability and impatience. She had also been driving recklessly, prompting her spouse to take away her car keys.

Elisa’s spouse also expressed concerns about her recent behaviour, including lying to friends about her accomplishments and borrowing money from family members under false pretence. She had also taken items from stores without paying, but she dismissed the incidents as “no big deal” when confronted about them.

When asked about her condition and how she explained this sudden change to herself, Elisa stated, “I just feel like I can finally see things clearly.” She denied hearing voices but mentioned feeling “guided” by something she couldn’t fully describe. Her spouse shared concerns about Elisa’s unpredictable behaviour and mood swings, which had caused tension at home.

ICD-10 Criteria

At least two affective episodes, one meeting the criteria for mania or hypomania.

Elisa met the ICD-10 criteria for a depressive episode when she first came into contact with psychiatric services. She presented with the following core depressive symptoms persisting for several weeks:

depressed mood: Reported by the patient and corroborated by her spouse, who described her as “tearful and unresponsive to attempts at reassurance.”

reduced energy leading to increased fatiguability and diminished activity. This was evident in her workplace, where she lacked motivation and would sit passively at her computer without engaging in tasks. Her spouse further described her as “increasingly withdrawn,” a behaviour likely linked to her reduced engagement with the external environment. (Withdrawal can also be interpreted as a consequence of *loss of interest and enjoyment*, another core symptom of depression.)

Furthermore, Elisa had the following additional symptoms of depression:

(b) *reduced self-esteem and self-confidence:* Described by the patient as feeling “inadequate.”

(e) *ideas or acts of self-harm or suicide:* She engaged in self-harm by cutting her thigh.

(f) *disturbed sleep*: She struggled to fall asleep and frequently woke up during the night.

(g) *diminished appetite*: Her appetite had declined, and she had lost 6 kg.

At her follow-up appointment, she had the following symptoms of hypomania or mania:

elevated mood: Characterised by a persistent feeling of being “confident” and “creative.” However, in line with ICD-10 diagnostic guidelines, elevated mood can also manifest as irritability, which in this case was described as moments of irritability and impatience.

increased energy: Described feeling “energised,”

increased activity: Engages in multiple projects, joins forums, exercises at night.

decreased need for sleep: Sleeps 3–4 hours/night without feeling tired.

increased sociability and overfamiliarity: Reconnects with acquaintances, shares personal stories inappropriately.

grandiosity: Claims to “see things clearly,” feels “guided.”

Diagnostic Considerations

Elisa meets the criteria for at least two affective episodes, first a depressive episode, followed by an episode characterised by elevated mood. While it can be debated whether the latter episode should be considered as mania or hypomania, this distinction is not essential for diagnostic purposes, as both qualify the patient for a diagnosis of bipolar affective disorder.

Certain traits described in her history may resemble features of a personality disorder, such as a tendency toward interpersonal conflict, impulsivity, and her own description of consistently feeling either “up” or “down,” which could be interpreted as affective instability rather than discrete mood episodes. However, it is unclear whether these traits are sufficiently pervasive or intense to meet the diagnostic criteria for a personality disorder. On the contrary, Elisa appears to function well overall, maintaining a stable job and family life. Most importantly, it is not possible to reliably assess a potential personality disorder during an ongoing affective episode.

In sum, the ICD-10 diagnosis of bipolar affective disorder is assigned here.

Case 6. Depressive Episode

Leonardo is a 24-year-old man. He came to the psychiatric outpatient clinic because he had difficulties thinking, concentrating, and remembering things. He also complained of being unable to sleep.

Leonardo was born and brought up in a peasant family in a small town. He was the fourth of seven siblings. His birth and development during childhood and adolescence were without complications. The atmosphere in his childhood home was good, and he described his family as warm and affectionate. At school, he was a good student, got good grades, and had several good friends. After completing his education, he moved to a city and got a job as an elementary school teacher. He lives in his own apartment and has a girlfriend, a 22-year-old nurse. They have known each other for 3 years and plan to marry next year. Leonardo’s girlfriend describes that he used to like to have fun, was easy going and sociable, with a lot of friends, and was interested in sports.

Leonardo’s difficulties began about 8 months ago, when he started having problems falling asleep and would lie awake for several hours every night before falling asleep. He would wake up at about 4 o'clock in the morning and not be able to fall back asleep. In bed, he would brood over all kinds of problems. Apart from that he felt as if “something was broken” in the middle of his brain, because he had been thinking too hard. By the time he came to the clinic, Leonardo was convinced that his brain cells did not work. As a result, he was not able to think, concentrate, or remember things, he said. His problem had gradually become so bad that he could no longer remember what he had read in the newspaper minutes before. At school teaching the pupils, he found himself unable to remember what he had been talking about in the previous class. Nevertheless, he forced himself to do his job.

He talked about what a failure he was, and how things he previously had found good were actually bad. He believed he had messed up everything for people around him, and that the agony he now felt was well deserved. He grew more and more miserable and could see no hope of changing his condition. He felt joyless and fatigued and reported that his appetite had been poor for 3 months and that he had unintentionally lost about 5 kg in weight. He no longer went out with his friends and family like he used to. He explained that he didn’t really feel like seeing them, and that he didn’t have the energy to do it.

Leonardo was appropriately dressed in blue jeans and a patterned sweatshirt. His face looked gloomy and anxious, with little expression. During the examination, he was polite and cooperative, always trying to answer the questions accurately. However, he was extremely restless and fumbled continuously with a newspaper that he had in his hands throughout the interview. He kept

returning to the idea that his brain was broken. He mentioned that he had contemplated suicide for a while, but that he had not made any plans for committing suicide.

ICD-10 Criteria

Core Symptoms:

depressed mood: Leonardo presents with a persistently low mood and describes feeling joyless and miserable.

loss of interest and enjoyment: He no longer engages in social activities with friends or family and has lost interest in things he previously enjoyed.

reduced energy leading to increased fatiguability and diminished activity: He reports feeling fatigued and lacking the energy to interact with others or carry out daily activities.

Additional Symptoms:

(a) reduced concentration and attention: Leonardo reports significant difficulties with thinking, concentration, and memory. He struggles to retain information from newspapers and his own teaching sessions.

(b) reduced self-esteem and self-confidence: He describes himself as a failure and believes he has negatively impacted those around him.

(c) ideas of guilt and unworthiness: Leonardo expresses the belief that his suffering is deserved and that he has “messed up everything”.

(d) bleak and pessimistic views of the future: He sees no hope for improvement and feels trapped in his current condition.

(e) ideas of self-harm or suicide: He has contemplated suicide.

(f) disturbed sleep: Leonardo experiences severe insomnia, including difficulty falling asleep, early morning awakening, and prolonged rumination in bed.

(g) diminished appetite: He reports a poor appetite and has unintentionally lost 5 kg over the past three months.

Diagnostic Considerations

Leonardo meets the ICD-10 criteria for a depressive episode. The onset of symptoms is clearly episodic, emerging about eight months ago. There is nothing that points to any psychiatric disorder or difficulties before the onset of the current episode. His belief that “something was broken in the middle of his brain” is best interpreted as a mood-congruent delusional idea within

the context of his depression. Thus, Leonardo's condition meets the ICD-10 criteria for a severe depressive episode with psychotic symptoms.

Case 7. Generalized Anxiety Disorder

Sofia is a 22-year-old woman. Seven months ago, she experienced two panic attacks. Initially, she believed she was suffering from a serious physical illness. At her general practitioner, she was informed that she was experiencing anxiety. Since then, she has avoided situations where she fears having another panic attack and where it is difficult for her to withdraw, such as cinemas, flights, and lectures.

She worries others will notice her distress and sometimes avoids social events due to this fear. She still fears having a serious physical illness and she also fears being alone with unfamiliar men. She experiences palpitations, choking sensations, dizziness, muscle tension, and feelings of unreality. She fears harming herself or her loved ones, and particularly serious illness. She sometimes struggles to fall asleep due to worries about dying during the night, but she sleeps well once asleep. Her appetite has decreased, and she has unintentionally lost 5 kg in the past six months.

She likes structure and considers herself very organized. She reports problems concentrating and slight memory deterioration over the past few months. She sometimes struggles to find the right words when speaking. It can be challenging for her to leave the dorm and interact with others in common areas as she does not always feel up to putting on a cheerful facade. She no longer enjoys going out with her friends the way she used to.

Sofia was born in the capital city and is the eldest of two siblings. Her mother experienced a depressive episode but was successfully treated with antidepressants. Her parents divorced when she was 9, initially resulting in her alternating weekly between their homes. The parents fought over custody, and the father's new wife was unkind to the children, prompting Sofia to cut off contact with her father at age 13. At around the same time, she experienced an attempted rape, but she managed to scare off the attacker by screaming.

She enjoyed school and had many friends. After 9th grade, she went to secondary school, where she was popular, socially active, and graduated with good grades. After secondary school, she worked as an au pair in Barcelona, Spain for a family and took Spanish lessons. She describes this period as the best year of her life, making many new friends and enjoying her time in Spain. The following year, she started education to become a social worker and passed the exams as expected. She has had several romantic relationships, lasting months to years, but she is currently single and lives in a student residence, which she enjoys. She is interested in the supernatural and recently consulted a clairvoyant.

She presents herself as engaged, initially smiling broadly but less so as the interview progresses. She is friendly, appears over-compliant, and slightly tense.

ICD-10 Criteria

(a) apprehension (worries about future misfortunes, feeling "on edge", difficulty in concentrating, etc.): Sofia fears serious illness, dying during sleep etc. She reports trouble concentrating, mild memory deterioration, and word-finding difficulties.

(b) motor tension (restless fidgeting, tension headaches, trembling, inability to relax): She explicitly reports “muscle tension” and has difficulty falling asleep due to persistent worry, indicating an inability to relax.

(c) autonomic overactivity

dizziness: Explicitly stated

tachycardia: Described as palpitations.

tachypnoea: Likely covered by what Sofia describes as ”choking sensations”.

epigastric discomfort: Not explicitly reported, but may be tentatively inferred from decreased appetite and unintended weight loss

Diagnostic Considerations

Sofia fulfils the diagnostic criteria for generalised anxiety disorder, with both psychological and autonomic components. She does not fulfil the ICD-10 criteria for panic disorder, which requires that *several severe attacks of autonomic anxiety should have occurred within a period of about 1 month* and *comparative freedom from anxiety symptoms between attacks.* Sofia, having had only two such attacks and experiencing many anxiety symptoms outside of these episodes, does not qualify for this diagnosis.

Agoraphobia is also not an appropriate diagnosis, as she does not fulfil the ICD-10 requirement that *anxiety must be restricted to (or occur mainly in) at least two of the following situations: crowds, public places, travelling away from home, and travelling alone.* Even though Sofia has had a tendency to avoid such places, her anxiety symptoms are not restricted to these, and she has too many other anxiety symptoms as well.

Sofia does not meet the criteria for hypochondriacal disorder. While she does fear serious illness, this concern is part of a broader anxiety profile that includes fear of panic attacks, social embarrassment, and being alone with unfamiliar men.

According to ICD-10, hypochondriacal disorder requires a *persistent refusal to accept the advice and reassurance of several different doctors that there is no physical illness or abnormality underlying the symptoms.* This feature is not present in Sofia, who readily accepted her general practitioner's explanation that her symptoms were anxiety-related.

Case 8. Obsessive–Compulsive Disorder

Olivia is a 19-year-old woman who has been referred to the community mental health team by her general practitioner. She has long been troubled by a series of intrusive thoughts that affect her daily activities and mood.

Initially hesitant to describe her life course, Olivia explains that she was born in a small city and lived with her family until the age of 8. Her father was depressed and had substance use disorder. At one point, she was hit by a car and broke her arm, because her father, who was supposed to be watching her, was drunk. She hasn't seen him since. Her mother found a new partner, and they moved to another part of the country. At her new school, Olivia was often angry and frequently had conflicts with the teachers. She changed schools and she did much better socially and academically at the new school. At 15, she started a college course to become a teaching assistant. She is now in her final year but plans to continue studying to become a nurse.

Olivia lives with her mother and stepfather. Her symptoms started at age 14, when she became obsessed with cleanliness, causing her to constantly wash her hands and change clothes. Her skin cracked due to eczema. Her condition has fluctuated a bit in the past four years. She still has issues with handwashing, doubting her cleanliness. She acknowledges that this behaviour is excessive. She is sometimes able to resist the thoughts about being dirty, which she finds unpleasant. However, her ritual activity currently takes up about an hour daily, mostly in the morning and evening.

Olivia sleeps a lot (9 hours at night and a 2–hour nap during the day). She believes that it is because she uses a lot of energy to suppress the thoughts during the day, e.g., she refrains from washing her hands during classes, despite thinking that her hands are dirty.

She denies experiencing anxiety now, but she admits having experienced it in the past. The last time she experienced anxiety was when she realised she had forgotten to return a Nintendo game to a friend, leading to sleepless nights, cold sweats, shaking, and distress. She thought that the friend would be angry and maybe even harm her or her family. However, she could still go to school, and eventually she distanced herself from these thoughts, which lasted for two days.

She has had similar experiences many times, usually involving guilt over trivial matters. She then gets ideas of how she will be punished. For example, she once worried about having lied to her mother when she was a child. These symptoms intensify for periods lasting up to one month. It usually happens once to twice a year.

During these times, she can attend classes but does not feel like herself as she is constantly thinking about these matters. After classes, she stays with her mother at work or at home,

because her mother can calm her down. She does not see her friends during these periods. She wakes up several times at night, immediately resumes thinking, and can be awake for a couple of hours. In these periods, she may feel that it would be easier if she were not here, but she has never made any plans for suicide. Her appetite is normal.

During the psychiatric interview, she appears nervous and quickly dismisses many questions, and it is unclear if they are entirely new to her or if she has difficulty reflecting on them. She struggles to structure the narrative of her life course, requiring the psychiatrist to continuously prompt her with many questions.

ICD-10 Criteria

Olivia has obsessional symptoms and compulsive acts that have been *present on most days for at least 2 successive weeks* and are a *source of distress or interference with activities.* These symptoms meet the following criteria:

(a) recognized as the individual's own thoughts or impulses: Not directly stated, but can be inferred from her recognition that these thoughts being “excessive,” and further supported by the absence of any evidence pointing to psychotic symptoms.

(*b) there must be at least one thought or act that is still resisted unsuccessfully, even though others may be present which the sufferer no longer resists*: She actively tries to resist washing her hands, though not always successfully, as she spends approximately an hour daily engaged in ritualistic behaviour.

(c) *the thought of carrying out the act must not in itself be pleasurable (simple relief of tension or anxiety is not regarded as pleasure in this sense)*: There is no indication that Olivia finds the compulsive acts pleasurable. Her behaviour appears driven by discomfort and the need to relieve distress, not by enjoyment.

(d) *the thoughts, images, or impulses must be unpleasantly repetitive*: Her intrusive thoughts about cleanliness and guilt are recurring and distressing. For example, her concerns about cleanliness led her to wash her hands so repetitively that she developed cracked skin from eczema, and she continues to worry about these thoughts even four years later.

Diagnostic Considerations

Olivia meets the ICD-10 criteria for obsessive-compulsive disorder, with clear examples of both obsessional thoughts and compulsive behaviours in response to the thoughts. These are distressing, repetitive, resisted, and interfere with her daily functioning.

Case 9. Emotionally Unstable Personality Disorder

Lucia is a 26-year-old woman referred to outpatient psychiatric services by her general practitioner, who initially attempted to treat her for depression. However, he subsequently determined that her condition required specialized psychiatric evaluation.

Lucia has recently completed her high school exams and is about to begin studies to become a dietitian. It took her additional years to complete her secondary education as even the smallest challenges overwhelmed her.

She grew up with her parents, who are both successful academics, and two elder siblings. None of them have had contact with psychiatry.

She went to a private primary school, which focused on creative subjects, but she felt out of place, academically superior to her peers, and she found school boring. She had two friends in this school. In year 10, she began experiencing sadness, anxiety, and insecurity.

In secondary school, she struggled with the studies, which didn't interest her much, and she found her classmates "uninspiring." Eventually, she dropped out of school. Later, she began taking individual school courses but only a few at a time.

Lucia reports that her mood and feelings usually change considerably during the day. Sometimes, she does not just feel happy but euphoric. She says she can become completely crazy with desire, unable to focus on anything else. She also describes often getting angry or annoyed when "other people disrespect me". She said, "I often feel really sad and angry. It's very hard to control those feelings, and they have sometimes led me to attempt suicide". Lucia has attempted suicide about six times. She also reports often feeling "empty in her heart" and has thoughts of being stuck and not fitting in.

Lucia explains that she might spend days in bed, but then experience "a sudden burst of energy" that leads her to start a project that she then carries on, often for several days. For example, replanting the entire garden or cleaning her parents' house, putting attention into every detail. She explains, "sometimes, I get really excited and quickly jump on new ideas. But there are also times when I feel very indecisive and need to talk to my friends and family before making any decision."

Lucia describes her sense of identity as "non-existent", and being in a new environment, such as an internship, causes her to change significantly. This includes altering her political opinions, the way she talks, and even how she dresses.

Socially, she has always felt out of place but adapts to any situation. She easily establishes relationships but has difficulties keeping them. She has one friend, whom she has known for many years, while her other acquaintances seem to come and go. She also describes that people in her

life have often disappointed her, and she then breaks off the relationship. Nonetheless, she rarely spends time alone, and if she is alone, she is usually on the phone with her friend. She has had several short-term relationships. She tends to return to a boyfriend after a breakup, leading to prolonged "on/off" periods. She describes that most of her boyfriends have had significant psychological difficulties. She has had many conflicts with her boyfriends, and she has acted violently against all of them. She has also threatened her mother and thrown things in anger.

Lucia smokes cannabis on rare occasions but does not drink alcohol.

ICD-10 Criteria

Lucia meets the general criteria for a personality disorder:

(a) markedly disharmonious attitudes and behaviour, involving usually several areas of functioning:

affectivity: Explicitly acknowledges to be strongly affected by extreme emotions.

impulse control: Suicide attempts in response to intense emotions, violence toward partners, threats, and throwing objects at her mother.

ways of perceiving and thinking: Lucia describes her identity as “non-existent” and reports shifting her political views and appearance in new environments. This instability in personal identity and internal preferences appears reflective of disturbances in ways of perceiving and thinking.
interpersonal relationships: Intense and unstable relationships with family members and romantic partners, and difficulty maintaining friendships.
(b) the abnormal behaviour pattern is enduring and long-standing, and not limited to episodes of mental illness: Lucia’s difficulties are not limited to isolated episodes like depression. Instead, they reflect a consistent pattern of emotional instability, impulsive behaviour, and troubled relationships. These traits show up regardless of her mood and persist even when she is not in crisis, suggesting a deeper, ongoing personality structure rather than symptoms limited to episodic mental illness.
(c) the abnormal behaviour pattern is pervasive and clearly maladaptive to a broad range of personal and social situations: Lucia’s behaviour has caused significant difficulties in her academic performance, interpersonal relationships, and emotional stability. She struggled to complete her education due to being overwhelmed by minor challenges, experiences intense and unstable relationships marked by conflict and impulsivity and displays marked emotional dysregulation.
(d) the above manifestations always appear during childhood or adolescence and continue into adulthood: Lucia’s symptoms began in adolescence, with emerging sadness, anxiety, and identity disturbance in the tenth grade, and have persisted into adulthood.

(e) the disorder leads to considerable personal distress but this may only become apparent late in its course: Evident in repeated suicidal behaviour, emotional crises, and chronic feelings of emptiness.

(f) the disorder is usually, but not invariably, associated with significant problems in occupational and social performance: There is extensive evidence of unstable relationships with family, romantic partners, and friends. Her education is significantly delayed, and there is no information indicating professional employment.

Lucia specifically meets the description of emotionally unstable personality disorder:

tendency to act impulsively without consideration of the consequences: Exemplified by several suicide attempts in response to intense emotional states.

affective instability: As described above, emotional dysregulation is so pronounced that it has led to multiple suicide attempts.

outbursts of intense anger may often lead to violence: Violence against romantic partners, throwing things at her mother.

She further qualifies for the Borderline subtype of emotionally unstable personality disorder:

Disturbance of *self-image, aims, and internal preferences:* Reflected in her description of her identity as “non-existent” and her tendency to change speech, opinions, and appearance depending on the environment.

chronic feelings of emptiness: Expressed as feeling “empty in her heart.”

intense and unstable relationships: Evident through repeated breakups, conflicts, and difficulty maintaining connections.

excessive efforts to avoid abandonment: Evident in her constant contact with a friend, discomfort when alone, and repeated return to romantic relationships despite their dysfunctional nature.

suicidal threats or acts of self-harm: Several suicide attempts are documented.

Diagnostic Considerations

It can be argued that Lucia, in addition to meeting the description of borderline subtype of emotionally unstable personality disorder, also meets the description of the impulsive subtype, given her pronounced impulsive features. However, differential diagnosis between subtypes of emotionally unstable personality disorder is not important for our purposes, as both subtypes were combined in all analyses.

She does not meet the criteria for bipolar disorder. Although she reports dramatic shifts in mood, such as feeling “not happy but euphoric” and “very sad”, these fluctuations are better understood as emotional reactivity rather than distinct affective episodes. There is no evidence of sustained depressive or hypomanic episodes lasting the required minimum duration (e.g., 14 days for depression, 4 days for hypomania). Instead, Lucia’s intense emotional states appear to be triggered by interpersonal stress such as feeling disrespected by others. Additionally, there is no indication of stable, well-functioning periods between these emotional shifts, which would be expected in an affective disorder.
